# Novel members of quinoline compound family enhance insulin secretion in RIN-5AH beta cells and in rat pancreatic islet microtissue

**DOI:** 10.1038/srep44073

**Published:** 2017-03-08

**Authors:** Z. Orfi, F. Waczek, F. Baska, I. Szabadkai, R. Torka, J. Hartmann, L. Orfi, A. Ullrich

**Affiliations:** 1 Department of Molecular Biology, Max-Planck Institute of Biochemistry, Martinsried, Germany; 2 Vichem Chemie Research Ltd., Budapest, Hungary; 3 Institute of Neuroscience, Technische Universität München, Biedersteiner Str. 29, 80802, Munich, Germany; 4 Department of Pharmaceutical Chemistry, Semmelweis University, Budapest, Hungary

## Abstract

According to clinical data, some tyrosine kinase inhibitors (TKIs) possess antidiabetic effects. Several proposed mechanisms were assigned to them, however their mode of action is not clear. Our hypothesis was that they directly stimulate insulin release in beta cells. In our screening approach we demonstrated that some commercially available TKIs and many novel synthesized analogues were able to induce insulin secretion in RIN-5AH beta cells. Our aim was to find efficient, more selective and less toxic compounds. Out of several hits, we chose members from a compound family with quinoline core structure for further investigation. Here we present the studies done with these novel compounds and reveal structure activity relationships and mechanism of action. One of the most potent compounds (compound 9) lost its affinity to kinases, but efficiently increased calcium influx. In the presence of calcium channel inhibitors, the insulinotropic effect was attenuated or completely abrogated. While the quinoline TKI, bosutinib substantially inhibited tyrosine phosphorylation, compound **9** had no such effect. Molecular docking studies further supported our data. We confirmed that some TKIs possess antidiabetic effects, moreover, we present a novel compound family developed from the TKI, bosutinib and optimized for the modulation of insulin secretion.

Tyrosine kinase inhibitors (TKIs) have proven anti-diabetic effect in different animal models and in clinical cancer patients as well^1^,^2^,^3^,^4^. How these TKIs could relieve diabetic symptoms is not completely understood yet. Their potential mechanism of actions leading to hypoglycemic effects have been recently summarized in several review papers^5^,^6^,^7^,^8^. According to the current knowledge the inhibition of c-Abl, PDGFR and VEGFR are considered as important factors in the remission of diabetes, nevertheless it does not give a full explanation for their mode of action. There are only a few studies available that investigated the direct effects of TKIs in beta cells. Reportedly, imatinib induced insulin secretion in the mouse pancreatic beta cell line NIT-1^9^. However in another study performed on human and rat pancreatic islets imatinib did not affect insulin secretion^10^. The latter observation is supported by other findings obtained with MIN6 mouse beta cells, mouse and human islets^11^. In contrast to imatinib, sunitinib was able to increase insulin level and decrease blood glucose level in a non-obese, spontaneously diabetic Torii rats animal model^3^. Based on these observations we hypothesized that insulin secretion could be directly stimulated by TKIs in beta cells. First we studied the effects of commercially available TKIs by using an insulin ELISA assay and found that some of them were able to induce insulin release in RIN-5AH beta cells. Because this cell line was responsive to various insulin secretagogue drugs (GLP-1, exenatide, glibenclamide and PDE4 inhibitors), it was chosen as a model for the studies of unknown compounds. Out of the 6 commercially available TKIs (sunitinib, imatinib, bosutinib, tivantinib, sorafenib and dasatinib) that we tested for insulin secretion, sunitinib was the most effective (Supplementary Figs 1 and 2). With the purpose of finding additional, more efficient and preferably less toxic candidates, we established a rationally designed compound library. The library consisted of 558 various molecules including the 6 commercial TKIs. Their kinase targets were known or predicted to overlap with the target profile of sunitinib. Most of the hit compounds could be classified into different groups according to their core structures. We identified strong hits with the following core structures: N-phenylpyrimidin-2-amine; 1,6-naphthyridine; quinoline; 5,6,7,8-tetrahydrobenzothiopheno[2,3-d]pyrimidine; quinazoline; 2-[(E)-styryl]quinazoline; indoline and quinoxaline. In this article we would like to report our results achieved with the quinoline derivatives only. After choosing this compound family for further investigation, additional derivatives were synthesized beyond the compounds included in the initial library. We demonstrate altogether 79 novel quinoline molecules in this paper that can be considered as derivatives of bosutinib, however they displayed notable differences in respect to insulin secretion and protein tyrosine phosphorylation. Interestingly, we found that minor modifications of the molecular structure unfolded an altered mechanism of action, which could be either based on the induction of calcium influx or tyrosine kinase inhibition. In this paper we demonstrate a structure activity relationship (SAR) analysis also that is necessary to interpret the transition from the TKI property towards the calcium influx inducer effect. Further on we focus on the characterization of the highly potent quinoline compound, **9** which induces insulin secretion in RIN-5AH cells and 3D rat pancreatic islet microtissues.

## Results

### Structure activity relationships (SAR) of quinoline derivatives

**Figure 1:**
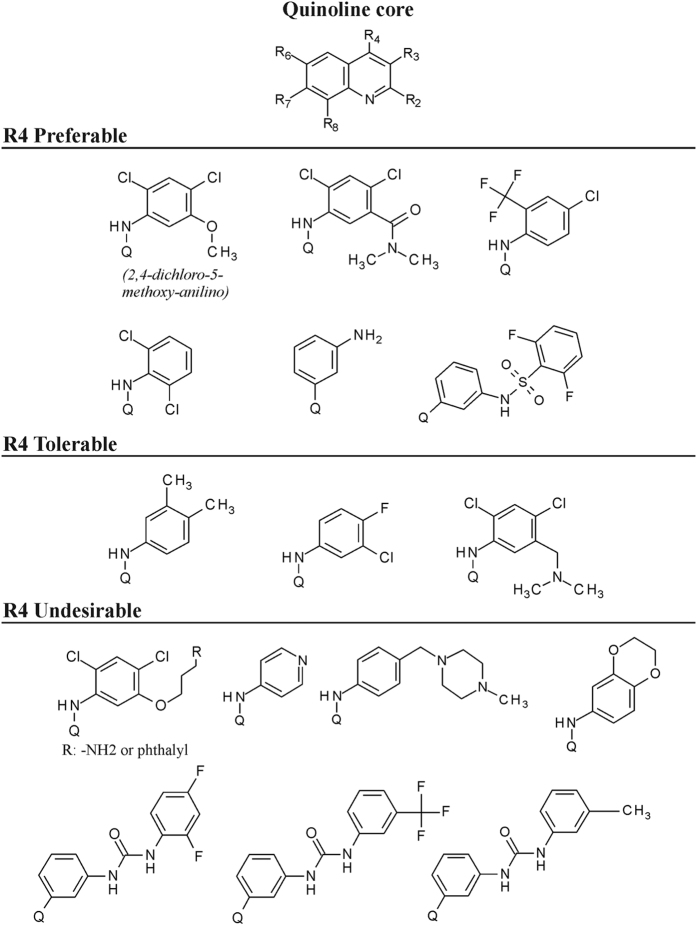
The quinoline core structure and examples of R4 substituents that differently influenced insulin secretion in RIN-5AH beta cells. Quinoline core structure is displayed at top. The three groups of R4 substituents are displayed below. “Preferable” represent a group of molecules where at least 4 derivatives with the same R4 substituent increased insulin secretion by at least 40% (first row) or if exclusive replacement of R4 group didn’t decrease insulin secretion of an efficient derivative (second row). “Tolerable” R4 groups don’t necessarily spoil the effect, depending on the R6,7,8 substituents. Third group represents derivatives with undesirable changes where if replacements carried out solely with any of the indicated R4 substituents spoiled the effect of its efficient derivative. Q denotes connection to quinoline ring through R4 position. For more details please see Supplementary Table 1.

In the primary screen there were 552 novel synthesized compounds and 6 commercially available TKIs included. We found that sunitinib produced a superior effect over the other 5 commercial compounds and showed a significantly improved insulin secretion over bosutinib as well (Supplementary Fig. 2). By further searching for additional active candidates in our screening setup, we achieved a 10% hit rate (data not shown). Out of these hits, the highly potent quinoline compound family was selected for further investigation. There were altogether 80 quinoline molecules tested for insulin secretion, including bosutinib that is sharing the same quinoline core (Supplementary Table 1). The prominent structural differences between bosutinib and these novel quinoline derivatives are the disposition of CN group from R3 to R2 (3-CN to 2-CN), moreover the replacement of R6 and R7 groups to smaller substituents e.g. OCH_3_ or F (compounds **8**, **9**, **10, 11**). These compounds produced 6–10 times stronger insulinotropic effect compared to bosutinib. The Br, F or CF_3_ functional groups in R8 position also contributed to the effect (compounds **7, 13, 14**), however, an addition of a F to R6 completely abolished the effect (compound **33**). The displacement of CN group from R2 to R3 significantly attenuated the effect (compound **15**). When OCH_3_ or F was present in R6 or R7 positions, the replacement of R2 CN groups to CONH_2_, methanesulfonamidomethyl, COOH, CH_2_NH_2_ or CH_3_ (compounds **17, 21, 22, 32, 34**) dramatically reduced the effect. The above described SARs of compounds, with the 2,4-dichloro-5-methoxy-anilino group at R4 position, are displayed in Table 1. Further important SARs could be specified when substituents were exchanged in R4 position. These substituents were classified into 3 groups according to their impact on the stimulation of insulin secretion measured by ELISA (Fig. 1).

**Figure 2:**
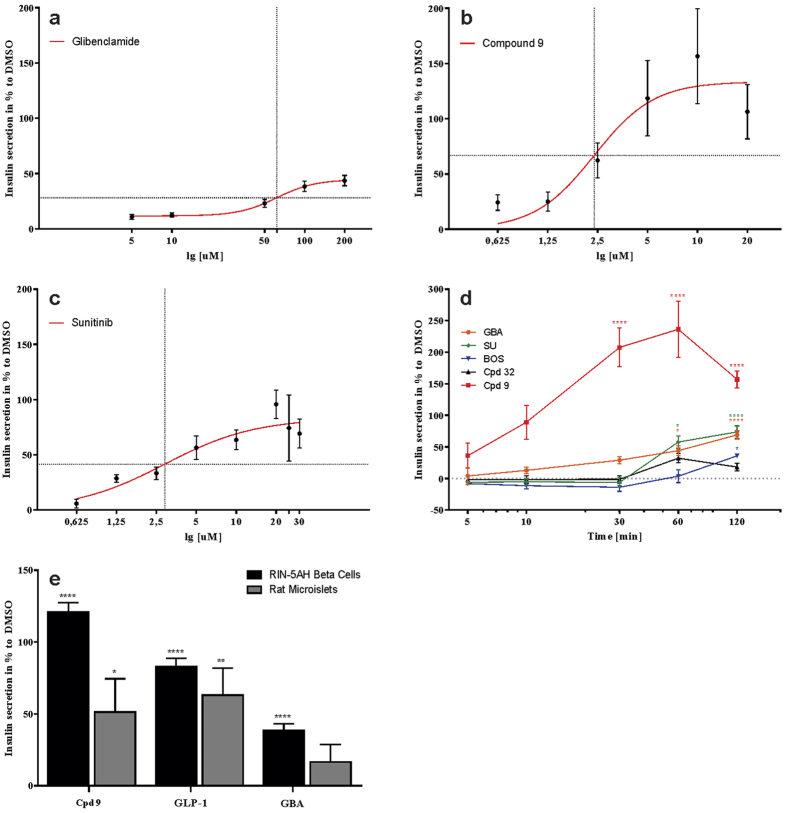
Concentration and time dependent insulin responses of beta cells upon the treatment with quinolines, GBA and bosutinib. Values are indicated in %, compared to DMSO treated cells (DMSO = 0%). (**a–c**) Graphs representing the insulinotropic effects of glibenclamide, compound 9 and sunitinib in RIN-5AH cells treated for 2 h. The EC50 value for compound 9 was detected at 2.38uM for GBA at 61.86 uM and for sunitinib at 2.89 uM. The maximal effect was much higher in the case of compound 9 (n = 4-8; SEM). (**d**) TKI compounds sunitinib and bosutinib had a different kinetics and their effects developed later. Compound 9 was able to significantly increase insulin secretion after 30 min compared to DMSO and to all other compound treatments at indicated time points (30 min, 60 min and 120 min; ****p < 0.0001). After 120 min treatment with glibenclamide (GBA) and sunitinib (SU) insulin secretion was significantly elevated (****p < 0.0001), bosutinib (BOS) treatment showed a lower level of significance (p = 0.0217), while a non-active quinoline analogue, compound 32, caused no significant insulin secretion (n = 4; SEM; ANOVA/Tukey). (**e**) Validation of insulinotropic effect of compound 9 (Cpd 9) and control compounds in rat islet microtissue and comparison to RIN-5AH beta cells. Cells and islets were treated for 2 h with 5 uM compound 9, 0.3 uM GLP-1 and 100 uM glibenclamide (GBA). Rat islet microtissues proved to be less sensitive, but compound 9 and GLP-1 showed significant increase in insulin secretion compared to DMSO (n = 4-8, SEM, t-test, *p < 0.05; **p < 0.01; ****p < 0.001).

For further tests, one of the most potent quinoline derivative was chosen (compound **9**). It stimulated insulin secretion in RIN-5AH beta cells in a concentration and time dependent manner. The EC50 was determined at 2.38 μM, whereas the maximal effect was observable between 10–20 μM. Glibenclamide (GBA) was used as a positive control, its EC50 was 61.86 μM and the maximal insulin response was detected at 100–200 μM. It is important to notice that the maximal effect of insulin secretion was much higher in case of compound **9** treatment. The EC50 of sunitinib was 2.89 μM (Fig. 2a–c). Kinetic measurements revealed quick acting characteristics for compound **9** and after 10 min treatment a significant insulin release was detected already. Other compounds like GBA, sunitinib, bosutinib and a non-secretagogue quinoline compound, **32** showed different kinetics and their on-set effects were observable at 60–120 min (Fig. 2d). The insulinotropic action of compound **9** was confirmed in rat islet microtissues. Compared to RIN-5AH cells, the islets seemed to be less sensitive not only to compound **9** but also to GLP-1 and GBA (Fig. 2e).

### Compound 9 is not targeting kinases

**Figure 3:**
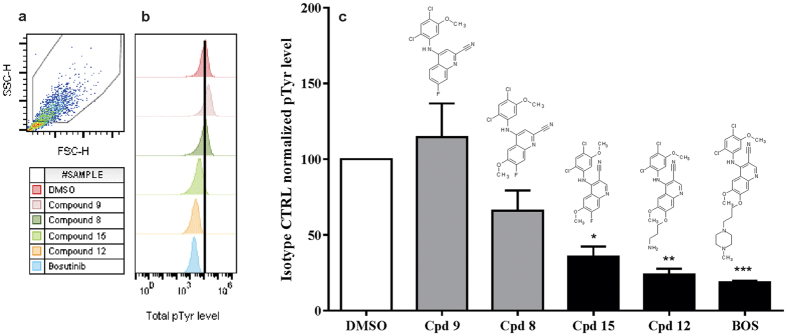
Total pTyr level in RIN-5AH beta cells, after treatment with various quinoline derivatives. (**a**) Gated cells for evaluation. (**b**) Representative graphs of fluorescence intensities measured in FL2-H. Fluorescence which is proportional to pTyr level is significantly reduced after treatment with compound 15, compound 12 and bosutinib, while compound 8 and compound 9 didn’t affect tyrosine phosphorylation. (**c**) Quantitative evaluation of total pTyr levels in RIN-5AH cells after treatment with quinoline compounds and bosutinib (BOS) at 5 μM for 2 h. Grey bars indicate treatments with 2-ciano-quinoline derivatives, black bars stand for 3-ciano-quinolne derivatives. Data is normalized to samples incubated with isotype control Ab and represented in % to DMSO (DMSO set to 100%). (n = 3; SEM; ANOVA/Tukey).

Results of the kinetic experiments already suggested a different mechanism of action for compound **9** compared to bosutinib and sunitinib. Bosutinib and sunitinib are reported to hit many kinases besides their main targets src, Abl and VEGFR, PDGFR, KIT, RET^12^,^13^. With the purpose to ascertain what targets could be different or common between sunitinib, bosutinib and compound **9**, the substances were tested against 392 non-mutant and 59 mutant kinases (386 non-mutants and 56 mutants in case of bosutinib) in a competitive binding assay under identical conditions. The selectivity panel was provided by DiscoverX (Fremont). Surprisingly we found that however the predicted target profile of compound **9** should overlap with the target profile of bosutinib or sunitinib, as the results are showing there were no high affinity binders identified for compound **9**. Besides that, sunitinib and bosutinib hit 219 and 147 non-mutant kinases respectively (Supplementary Fig. 3). Next, we selected bosutinib and some of the intermediate derivatives to confirm non-tyrosine kinase inhibitory activity and to find out the SAR how total tyrosine phosphorylation (pTyr) is affected by the different substituents on the quinoline ring. The pTyr level was significantly reduced after treatments with bosutinib, compound **12** and compound **15**, on the other hand for compound **8** and compound **9** this was not the case. Compound **9** differs from compound **8** only by lacking the 6-methoxy group (6-OCH_3_) on the quinoline ring. A nonsignificant but moderately reduced tyrosine phosphorylation caused by compound **8** suggested that the 6-OCH_3_ might also play a role in targeting kinases, because the removal of this group seemed to further impair the TKI effect (Fig. 3). Results of antibody controls are indicated in Supplementary Fig. 4. The markedly reduced TKI effect caused by compound **9** was confirmed by western blotting. Furthermore, no changes were visible in phospho-serine nor in phospho-threonine levels by western blotting (Supplementary Fig. 5).

### Docking simulations with Abl and Src kinases in Schrodinger Suite suggest a non-TKI property for compound 9

**Figure 4:**
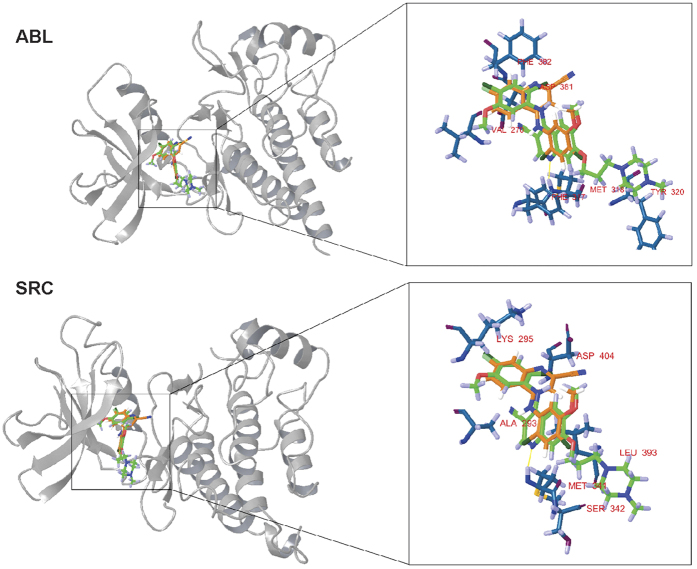
Docking poses of compound 9 (orange) compared to bosutinib (green) on the ATP binding site of Abl and Src. H-bonds are marked with yellow lines. Bosutinib forms hydrogen bond with the hinge region of both kinases (Abl – Met318; Src – Met341).

Compound **9** lost its affinity to Abl and Src kinases, which are main targets of the quinoline TKI, bosutinib. Although compound **9** and bosutinib share the same core structure, they have a differently positioned nitrile group (CN) on the quinoline ring as mentioned above. It was reported that this R3 CN group in bosutinib played an important role in the binding to Abl kinase^14^. By examining the binding mode of bosutinib, hydrogen bonds could be observed with the hinge region of both kinases (Fig. 4). In the case of compound **9**, Glide did not found any hydrogen bonds with the ATP binding sites and the docking scores (Abl: −6.896 kcal/mol, Src: −6.362 kcal/mol) were only moderate as well. These results are in good correlation with the biochemical data, as compound **9** does not inhibit Abl nor Src kinases.

### Quinoline derivatives induce Ca^2+^ influx in RIN-5AH beta cells

**Figure 5:**
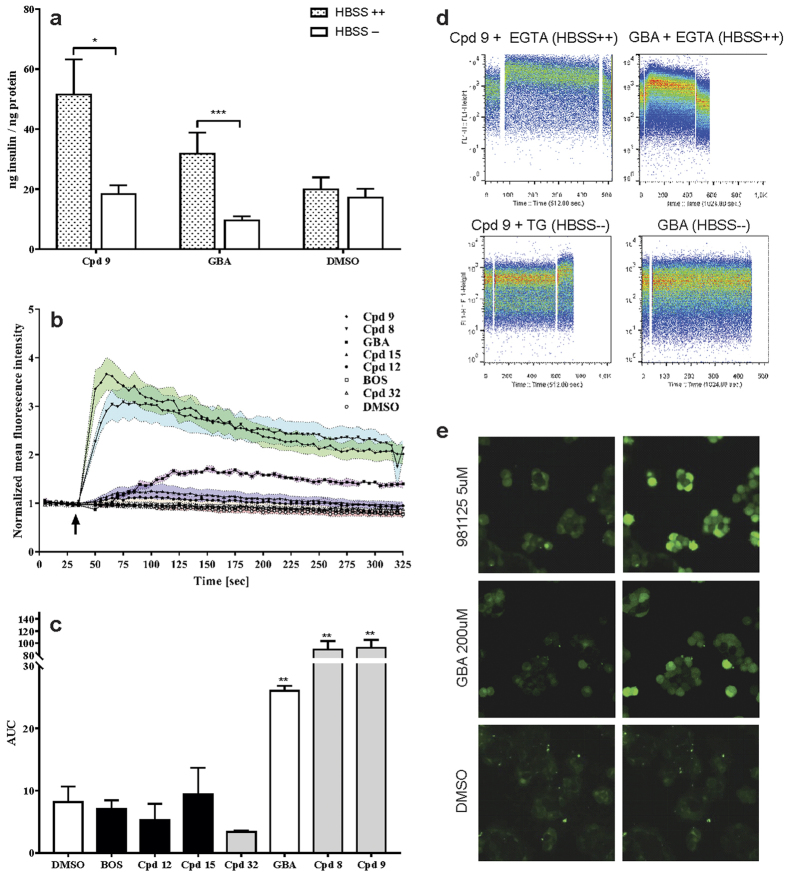
The mechanism of action of Compund 9 is linked to extracellular calcium influx. (**a**) RIN-5AH cells secreted significantly lower amount of insulin in HBSS− compared to HBSS++ buffer when treated with compound 9. Glibenclamide (GBA) had no effect in HBSS− either (5μM for VCC981125 and 100μM for GBA; n = 4–8; SEM; t-test; *p < 0.05; **p < 0.01; ****p < 0.001). (**b**) Calcium influx was monitored on FACS. Graph displays the changes of [Ca]_i_ in Fluo-4AM loaded RIN-5AH beta cells. Arrow indicates the addition of compounds (at 35 sec). Each data point represents the mean of a 5s gate. Outer dashed line represents borders of standard error (200 μM for GBA and 5 μM for other compounds; n = 3–5) (**c**) Quantitative evaluation of calcium influx representing the AUC (area under the curve) values. The baseline was defined from 0-35sec and the area was calculated from 50-325 sec. Grey bars indicate the 2-ciano-quinoline derivatives, black bars the 3-ciano-quinolne derivatives and white bars the negative (DMSO) and positive (GBA) controls (n = 3–5; SEM; t-test). (**d**) Representative graphs illustrating calcium influx measured on FACS. A gap denotes the addition of compounds to the cells. Compound 9 and GBA increased calcium influx in Ca2+/Mg2+ containing, but not in Ca2+/Mg2+ free buffer. The signal proportional to free Ca2+ level could be quenched by using EGTA in both cases. Thapsigargin (TG) was used as positive control to validate the experiment. It induced calcium release from internal Ca2+ stores in HBSS−. (**e**) Images showing the Fluo-4 loaded RIN-5AH beta cells treated by various compounds. First column represents cells before treatment, second column after treatment.

Elevated intracellular calcium concentration ([Ca^2+^]_i_) is one of the most important secondary messenger mechanism that triggers the exocytosis of insulin vesicles in beta cells^15^. It was shown that kinases most likely did not play a role in the mode of action of the tested 2-CN substituted quinoline derivative compound **9**. By this we hypothesized that another mechanism was responsible and investigated if calcium influx was affected. Compound **9** and GBA treated RIN-5AH cells were tested for insulin secretion in HBSS buffer with or without Ca^2+^/Mg^2+^ (HBSS++or HBSS−). Neither GBA nor compound **9** could stimulate insulin release if HBSS− buffer was employed. These results denoted a calcium dependent mechanism. In our experiments, compounds with tyrosine kinase inhibitory property and characterized by a CN group in R3 position were not able to increase calcium influx (compounds **12, 15** and bosutinib), conversely the other two 2-CN derivatives (compound **8** and **9**) showed positive results. The impacts of compound treatments on Ca^2+^ influx are illustrated in Fig. 5. All treatments were done at 5 μM, except for GBA where 200 μM was applied. Additional reference compounds were also used in the assay. Quinidine, chloroquine, mefloquine (quinoline compounds) and the fluoroquinolone drugs gatifloxacin and levofloxacin were described to have an inhibitory effect on K_ATP_ channels which may lead to membrane depolarization and subsequent increase of [Ca^2+^]_i_ in beta cells^16^,^17^,^18^,^19^. In RIN-5AH beta cells the insulin secretion was stimulated by quinidine and chloroquine at 50 μM but not by fluoroquinolones. The calcium influx caused by quinidine was only detectable at much higher concentrations (Supplementary Figs 6 and 7). These results clarified that the primary mechanism of compound **9** was linked to the opening of calcium channels located in the plasma membrane and was not dependent on the mobilization of Ca^2+^ from the internal calcium stores. Evidently compound **9** was more effective compared to GBA, quinidine or chloroquine in respect to insulin secretion and calcium influx.

### Compound 9 induces membrane depolarization in RIN-5AH beta cells

**Figure 6:**
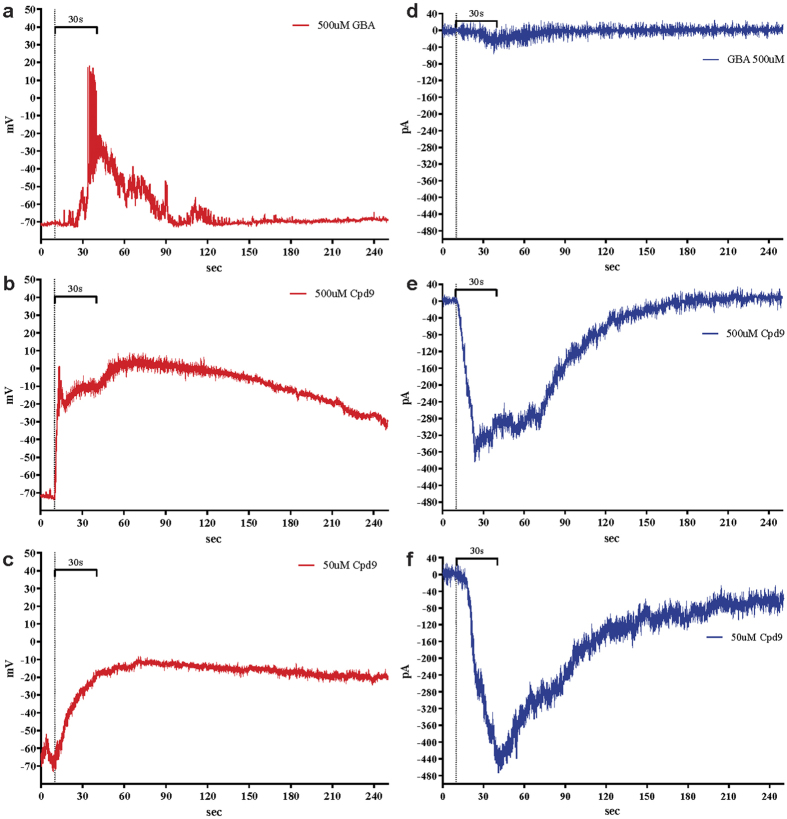
Representative graphs of patch clamp experiments performed in RIN-5AH cells after stimulation with glibenclamide (GBA) or Compound 9 (Cpd 9). Vertical dotted lines at 10 s indicate the starting timepoint of stimulation, which lasted for 30 s in each case (marked with horizontal lines). (**a–c**) Current clamp measurements after stimulation. (**d–f**) Voltage clamp measurements after stimulation. Compound 9 (Cpd 9); glibenclamide (GBA). Measurements were repeated at least three times on independent cells and gave similar results.

Membrane depolarization or other stimuli can induce and regulate the opening of voltage dependent calcium channels (VDCC) which can subsequently lead to increased [Ca^2+^]_i_ in the cells^20^,^21^. We used whole cell patch-clamp recordings to check if the calcium influx induced by compound **9** was a result of membrane depolarization. In our experiment a shift in membrane potential from −70 mV up to −20 mV (50 μM) or 0 mV (500 μM) was registered in current clamp (CC) mode. In cells treated with GBA (30 s; 500 μM) the depolarization was transient (approx. 90 s). The analogous application of compound **9**, in contrast, caused a sustained depolarization lasting at least for the duration of the recording (240 s; n = 3). The same difference in the kinetic properties of the responses to GBA and compound **9**, respectively, were observed in voltage-clamp recordings. The current intensity returned to basal value after 30 s in case of GBA, while in case of compound **9** it took approximately 3 min (Fig. 6). Furthermore, stimulation with compound **9** caused an immediate increase in inward current, unlike GBA where a short delayed response was registered, which could also refer to some differences in their mechanism of action. Interestingly, lower and higher concentrations of compound **9** (50–500 μM) showed comparable effect on inward current in VC mode (−400–500 pA), which in comparison to GBA (−40 pA) was apparently higher. A change in membrane capacitance was also detectable during compound stimulation, which indicated that exocytotic events happened in the cells^22^ (data not shown).

### Insulinotropic effect of compound 9 is dependent on Ca^2+^ and K^+^ currents in RIN-5AH beta cells

**Figure 7:**
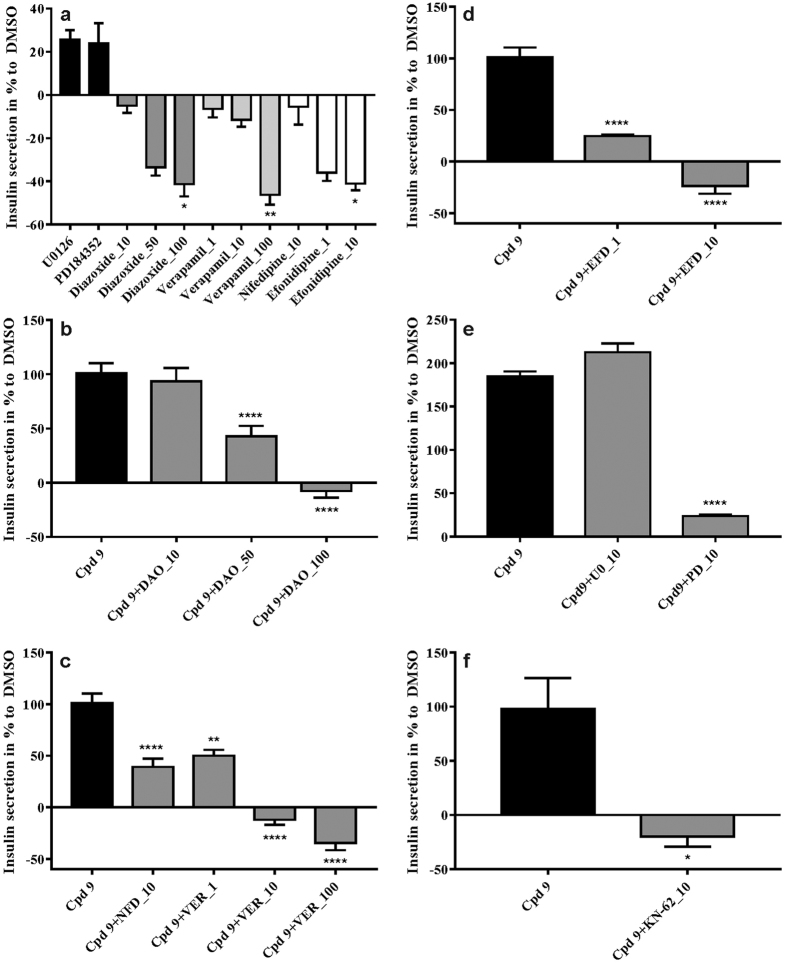
Combinatorial treatments with Cpd9 and ion channel modulators and their effects on insulin secretion in RIN-5AH cells. (**a**) Single compound treatments with ion channel modulators and MEK inhibitors. DMSO treatment was taken as a reference for calculating the significance. (**b**) Combination of compound 9 (5 μM) with KATP opener drug, diazoxide (DAO, 10;50,100 μM), (**c**) with L-type Ca2+ channel inhibitor, verapamil (VER, 1;10;100 μM) and nifedipine (NFD, 10μM), (**d**) with L-/T-type channel inhibitor efonidipine (EFD, 1 μM;10 μM), (**e**) with MEK inhibitors, U0126 (U0, 10μM) and PD184352 (PD, 10 μM) and (**f**) with CAMKII inhibitor KN-62 (10 μM). For (**b–f**) the significance is displayed taking Cpd9 treatment as the reference. (n = 4; SEM; 1-way ANOVA; *p < 0.05; **p < 0.01; ***p < 0.001; ****p < 0.0001).

To further investigate the mechanism of action of compound **9**, combinatorial treatments were performed by using small molecular inhibitors that interfere or block Ca^2+^ or K^+^ currents. Additionally, combination with inhibitors of MEK-ERK pathway and the CAMKII protein were also tested, since compound **9** upregulated ERK and CAMKII protein phosphorylation in RIN-5AH cells as well (see western blot results). Ion channel modulators that negatively affect insulin release were used in combination with compound **9**. High concentrations of single treatments with diazoxide (DAO), verapamil (VER) and efonidipine (EFD) were able to decrease insulin secretion in RIN-5AH beta cells significantly (Fig. 7a). By applying excessive amount of the K_ATP_ opener drug DAO (50 μM and 100 μM), it was able to reduce the concentration of secreted insulin in compound **9** treated cells. (Fig. 7b). Combinatorial treatments with L-type Ca^2+^ channel blockers, verapamil (VER) and nifedipine (NFD) resulted in a decreased or abrogated insulin secretion. (Fig. 7c). Applying 1 μM of the L-and T-type channel blocker efonidipine (EFD) significantly reduced and at 10 μM it completely abolished the effect of compound **9** (Fig. 7d). We used two types of MEK inhibitors (PD184352 and U0126) in combination with compound **9** to see how insulin secretion is altered if compound **9** induced ERK activation is blocked. Interestingly they produced different results. It was described by others that U0126 had the ability to block calcium influx and therefore hinder amino acid induced insulin secretion^23^. We were curious to see if it is also able to block the effect of compound **9**. We found that PD184352 abrogated the insulinotropic effect of compound **9** while U0126 had no such effect (Fig. 7e). The insulinotropic action of compound **9** was abolished by KN-62, an allosteric CAMKII inhibitor, which might additionally inhibit potassium channels too^24^ (Fig. 7f). The effects of GLP-1 and sunitinib were also investigated by combination treatments. The results suggested a distinct mechanism of action for compound **9**, as neither VER nor NFD were able to reduce the effect of GLP-1 and sunitinib significantly, indicating that they primarily stimulated insulin secretion without the involvement of L-type Ca^2+^ channels (Supplementary Fig. 8).

### Compound 9 activates CAMKII and ERK1/2 in RIN-5AH beta cells

**Figure 8:**
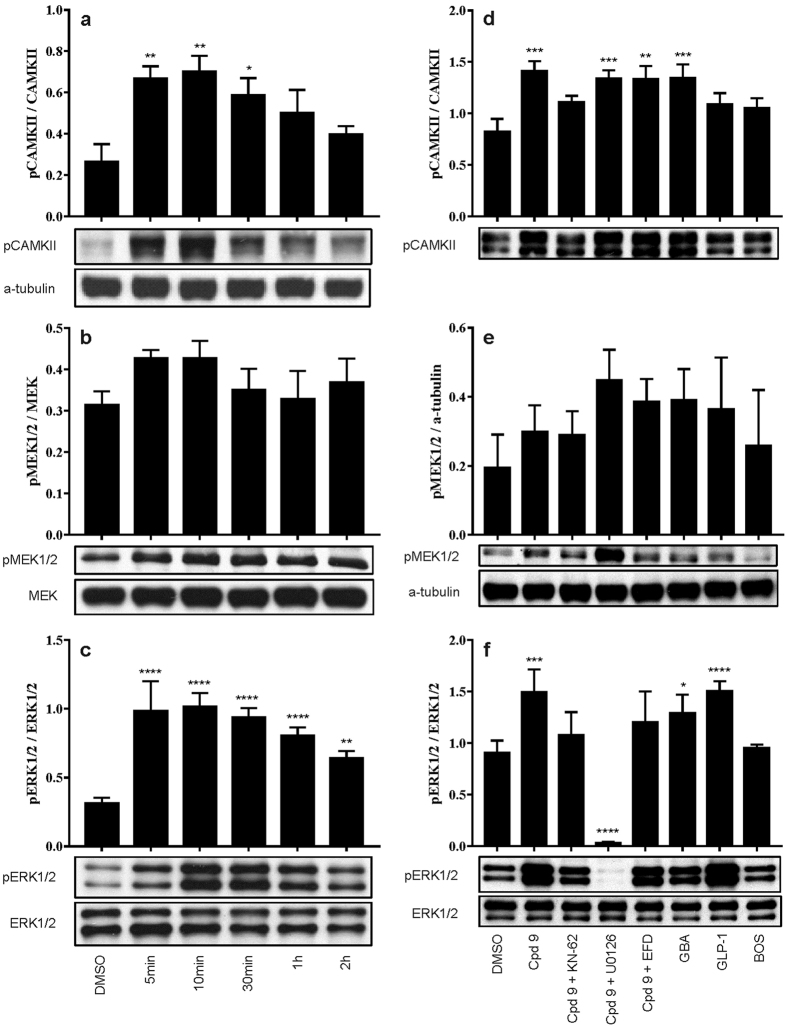
Investigation of the involvement of CAMKII, MEK1/2 and ERK1/2 in compound 9 stimulated insulin release. (**a–c**) Western Blot results of time dependent treatment. Cells were treated with 5 μM compound 9 for the indicated time. ERK1/2 and CAMKII were rapidly phosphorylated after treatment. MEK1/2 protein was slightly upregulated, however the change was not significant. Graphs are representing the quantitative results of western blot experiments. (**d–f**) Western Blot results of combination treatments in RIN-5AH cells. 5 μM compound 9 (Cpd 9) increased CAMKII and ERK1/2 phosphorylation. 10 μM KN-62 could block the stimulatory effect of cpd 9 on CAMKII. It reduced ERK1/2 phosphorylation as well, but didn’t affect pMEK1/2 level. 10 μM U0126 didn’t change phosphorylation state of CAMKII but caused a hyperactivation of MEK1/2, while completely blocked ERK1/2. 1 μM efonidipine was enough to reduce pERK1/2 level, but interestingly CAMKII could be still stimulated by cpd 9. 200 μM GBA upregulated CAMKII and ERK1/2. 0.3 μM GLP-1 involved ERK1/2 but not CAMKII phosphorylation. 5 μM bosutinib didn’t affect pCAMKII and pERK1/2 levels. Data are normalized to total ERK1/2 (for pERK1/2) or a-tubulin (for pMEK1/2 and pCAMKII). Experiments were performed three times and gave similar results. Data was normalized to total protein amount. Significance is shown compared to DMSO treatment (n = 3–4; SEM; Two way ANOVA/Dunnett).

Exocytosis and calcium influx related signaling pathways were checked by western blot studies. The calmodulin-dependent kinase II (CAMKII) is known to play a major role in mediating the effects of Ca^2+^ in the cells and was described to take part in the regulation of insulin secretion as well. Its activation correlated with insulin secretion and proved to have an important role in the regulation of glucose dependent insulin secretion in beta cells^25^,^26^,^27^. It is also known that treatments with various insulin secretagogue drugs (e.g. glibenclamide) involve ERK1/2 phosphorylation that may also correlate with an increased insulin secretion^28^,^29^,^30^,^31^. The activation of ERK1/2 protein can be further beneficial to beta cells by promoting cell survival, which was described in a recent study with imatinib^32^. Time dependent treatments were performed with compound **9** in RIN-5AH cells and results were analyzed by western blot. Short term treatments showed evidence for activation of calcium influx through upregulation of CAMKII and ERK1/2. It was also noticeable that protein phosphorylation and upregulation preceded insulin secretion, therefore the activation of CAMKII and ERK1/2 proteins could be a part of an early mechanism of the insulin response triggered by compound **9** (Fig. 8a–c). Combination treatments with compound **9** and CAMKII, MEK and calcium channel inhibitors were analyzed by western blot too. Pre-treatment with the CAMKII inhibitor KN-62 prevented the compound **9** induced activation of both ERK1/2 and CAMKII. The phosphorylation of ERK1/2 but not CAMKII was completely blocked in the presence of the MEK inhibitor U0126. Besides that, a strong MEK1/2 hyperactivation was observable. This phenomenon is known and was described in other *in vitro* studies using MEK inhibitors or siRNAs targeting the MEK-ERK pathway^33^,^34^. Applying 1 μM of the L- and T-type calcium channel blocker efonidipine prevented ERK1/2 but not CAMKII phosphorylation in our experiments. The compound induced upregulation of ERK1/2 could be also a result of GPCR activation therefore GLP-1 was included as a control compound^35^,^36^,^37^. For further controls glibenclamide (GBA) and bosutinib (BOS) were used. As expected, GBA induced the phosphorylation of CAMKII and activated ERK1/2 via the increased calcium influx. GLP-1 upregulated ERK1/2 but not CAMKII. Bosutinib did not affect CAMKII or ERK1/2 phosphorylation (Fig. 8d–f). These experiments showed that ERK1/2 and CAMKII are important signaling molecules transmitting the insulinotropic effect caused by compound **9**. Furthermore they highlight additional differences in the mode of action compared to glibenclamide, GLP-1 and also to bosutinib.

### Most novel quinoline compounds and compound **9** do not affect cell viability

The compounds used in insulin ELISA assays were tested with regard to their long-term effect on cell viability in RIN-5AH cells since the doubling time of these cells is more than 24 h. The main purpose of these experiments were to exclude the possibility of toxicity, e.g. stress induced insulin secretion^38^. The quinoline compounds, commercial TKIs and other inhibitors that were used in our experiments were tested by CellTiter-Glo. We selected staurosporine as a positive control in the assay, which induced strong cell death at 5 μM after 72 h. Other TKIs reduced the cell viability by 4–24%. From the tested set of quinolines only 12 compounds reduced the cell viability by more than 25%. Calcium channel inhibitors and the MEK inhibitor U0126 did not affect cell health. Another MEK inhibitor PD184352 disturbed cell viability, therefore U0126 was used in other experiments in combination with compound **9**. We noticed that high concentration of GBA (200 μM) also showed a high rate of toxicity in the cells. Results of the 72 h cell viability assay can be found in Supplementary Table 2. Dose response measurements were performed with compound **9** and we found that cell viability was affected only above 10 μM after 72 h incubation. We also investigated the effect of compound **9** on the proliferation in RIN-5AH cells by utilizing BrdU and propidium iodide (PI) + annexin V (AN) double staining assays. These time dependent treatments further support our findings that the compound **9** stimulated insulin secretion was not a consequence of a toxic effect (Supplementary Fig. 9a–d).

## Discussion

Our initial studies had been focused on the investigation of TKIs, however later on the results lead to the discovery of a novel compound family acting via a different mechanism. We demonstrated that structural modification of the TKI bosutinib made it possible to optimize and develop new compounds for the stimulation of insulin secretion.

By our screening approach, we identified potent TKIs and a novel quinoline compound family that significantly increased insulin secretion in RIN-5AH beta cells. We observed that the TKIs mainly targeting c-Abl (imatinib and bosutinib) were associated with a less prominent insulinotropic effect and sunitinib was the most potent amongst them. Although bosutinib and sunitinib share common kinase targets, in order to identify the relevant (and most likely a combination of) ones that play role in the enhancement of insulin secretion, would need more investigation. From the quinoline family, compound **9** proved to be more potent than sunitinib, hence it was chosen for further characterization. In the kinase affinity assay, 219 hits were identified for sunitinib, 147 for bosutinib and no hits were found for compound **9** by using the same cutoffs and concentrations. This result was confirmed in further experiments and a distinct mechanism of action involving calcium influx could be assigned to compound **9**. It is important to notice that increased [Ca]_i_ was only detectable in the presence of extracellular calcium, thus calcium release from intracellular calcium stores were primarily not part of the mechanism, more importantly extracellular calcium was necessary to exert its insulinotropic effect on RIN-5AH beta cells.

By analyzing structure activity relationships based on insulin secretion data and pTyr inhibition, we discovered that the disposition of R3 CN group to R2 mitigated the tyrosine kinase inhibitory effect, increased insulin secretion and caused calcium influx in RIN-5AH beta cells (compound **15** vs. **8**). Furthermore, the TKI effect completely disappeared when OCH_3_ was removed from R6, while the calcium influx effect was still preserved (compound **8** vs. **9**). The docking simulations also suggested that kinases are not hit by compound **9** and the R2 positioned CN is crucial for forming H-bonds between protein tyrosine kinase targets (e.g. Abl and Src) and the molecule.

Next, patch clamp experiments showed that compound **9** was able to depolarize the cell membrane in RIN-5AH cells and therefore voltage dependent Ca^2+^ channels are likely to be involved in its mechanism of action. Both GBA and compound **9** depolarized the cell membrane of RIN-5AH beta cells, however compound **9** induced a much stronger and longer lasting effect, furthermore a different kinetics of insulin secretion was detected. By using ion channel modulators in combination with compound **9**, we showed that its insulinotropic effect was dependent on K^+^ and Ca^2+^ current. By performing comparative combinatorial treatments with GBA, GLP-1 and sunitinib, we found that compound **9** had a distinct mechanism of action compared not only to TKIs but to GBA and GLP-1 as well. Namely, GLP-1 and sunitinib induced insulin secretion were not sensitive to verapamil or nifedipine in contrary to compound **9**. This differential analysis further helped us to confirm that GLP-1 receptor and TKIs were primarily not involved in its mechanism of action. It is further noticeable that GBA is known to be a sulfonylurea drug with very high affinity to both rat and human K_ATP_ channels^39^, in our experiments it showed a weaker effect on insulin secretion compared to compound **9**. Both RIN-5AH beta cells and pancreatic microislets were less sensitive to GBA treatments and a 20–40x higher concentration was necessary to register a significant insulin release effect. DAO could also not attenuate the secretagogue effect of GBA in RIN-5AH cells, which might be due to the low sensitivity of the cells.

Compound **9** induced the phosphorylation of CAMKII and ERK1/2, which could be a consequence of the increased calcium influx. The upregulation of ERK1/2 can be beneficial and might support the survival of beta cells similarly to GLP-1^36^. The reduced pERK1/2 level evoked by pre-treatments with KN-62 and EFD, correlated with a lower insulin secretion. In spite of ERK1/2 was downregulated by U0126, the compound **9** induced insulin secretion was still noticeable, in contrary to another MEK inhibitor, PD184352. These inhibitors affected cell viability in a different manner, thus this might serve as an explanation for the dissimilar insulin secretion data. It was also described in the literature by others, that PD184352 produced cytotoxic effect *in vitro* (Schwannoma cell lines) and also induced apoptosis in contrast to U0126^40^. In our experiments we also observed a significantly decreased viability by treatment with PD184352, but not U0126 (Supplementary Table 1). We hypothesize that off-targets of these MEK inhibitors could also play role and more investigation would be needed to clarify this phenomenon. Our western blot results suggest that both active ERK1/2 and CAMKII were necessary for compound **9** to exert its insulinotropic effect, furthermore its potency is dependent on Ca^2+^ and K^+^ currents. Taken into consideration that compound **9** has a quick acting characteristics, it is most likely targeting ion channel(s) or acting on their direct regulators. We think that it is not targeting the K_ATP_ channel and has a different mode of action compared to GBA, since the kinetics and efficiency was not comparable. Finally we tested the effect of compound **9** on cell viability and based on our results we found that the insulinotropic action is not in correlation with toxicity or stress induced secondary effect.

In summary, we discovered and developed novel quinoline derivatives from the TKI, bosutinib that were able to stimulate insulin secretion in RIN-5AH cells. Next, we characterized a highly potent member of this family that exerted its effect through membrane depolarization and calcium influx and confirmed by SAR that it is not acting on tyrosine kinases. In order to elucidate the exact molecular mechanism of membrane depolarization and how calcium influx is induced by compound **9**, further investigations are needed.

## Materials and Methods

### Compound library

The initial compound library consisting of 558 compounds used in the primary screen was selected from the NCL library (Nested Chemical Library) of Vichem Chemie Ltd. The compounds and the synthetic routes are described in our patent EP100064, with the application number: 16170166.9–1466. Sunitinib, imatinib, bosutinib, tivantinib, sorafenib and dasatinib were also provided by Vichem Chemie Ltd.

### Cell culture

RIN-5AH insulinoma beta cell line was cultivated in RPMI 1640 supplemented with 10% FBS, 2 mM glutamine (Life Technologies) and grown at 37 °C, 5% CO2 in a high humidity atmosphere. 3D InSight™ Rat Islet Microtissues were purchased from InSphero and were propagated according to user’s manual.

### Insulin ELISA

RIN-5AH beta cells were seeded in 96 well plates (4 × 10^5^) and grown overnight (ON). Cells were washed 1x with PBS and treated in medium or HBSS buffer with or without Mg^2+^ and Ca^2+^ (HBSS++ and HBSS−). Standard conditions were 5 μM and 2 h if not otherwise indicated. For the combinatorial treatments, compounds were mixed and cells were treated for 2 h. The pre-plated 3D InSight™ Rat Islet Microtissues (Insphero, Switzerland) were washed 2x in KRBH containing low glucose (131 mM NaCl, 4.8 mM KCl, 1.3 mM CaCl_2_, 25 mM HEPES, 1.2 mM KH_2_PO_4_, 1.2 mM MgSO_4_, 2.8 mM glucose) and pre-incubated for 2.5 h at 37 C, 5% CO_2_ in KRBH with low glucose. Treatments were performed in KRBH containing high glucose (16,8 mM). After treatments, sample supernatants were assayed immediately or stored at −20 °C until measurement. Dilutions were made if necessary then ELISA was performed according to the user’s manual (Mercodia, Sweden). Plates were read on BioTek Elx800 Absorbance reader. Secreted insulin level was normalized to total protein amount in RIN-5AH cells. Protein concentration was determined by BCA assay kit (Thermo Scientific) according to user’s manual. In microislets the secreted insulin amount was normalized to total insulin. Microislets were lysed using KRBH containing 1,25% TX-100.

### Docking simulations

The docking calculations were carried out using Schrödinger^®^ Suite 2009 update 2 molecular modeling program packages (Glide, LigPrep, Maestro, Protein Prep. Wizard). We used the previously described crystal structure of Abl (3UE4) and Src (4MXO) from the RCSB Protein Data Bank. Protein Preparation Wizard was applied to add hydrogens to the protein residues, to remove the water molecules from the crystal structure, to assign bond orders and to treat the missing disulfide bonds. The 3D structures of the ligands were prepared using LigPrep module with OPLS_2005 force field (epic, pH = 7.4). The described ligands in the PDB entry were chosen for receptor model generation. For the docking we used Glide in standard precision (SP) mode. The best conformations were saved for each ligand and false conformations were rejected by the energy filters.

### Determination of total pTyr levels by Flow Cytometry

1 × 10^6^ RIN-5AH beta cells were seeded in 6 well plate and incubated overnight. After washing once with PBS, they were treated with compounds at 5 μM and incubated for 2 h at 37C 5% CO_2_. Cells were washed with ice cold PBS then trypsinized on ice. After 3 min incubation samples were collected, centrifuged and resuspended in 750ul PBS. Cells were fixed by adding 1 ml 4% formaldehyde solution and incubated for 10 min at 37C. Samples were placed on ice, then centrifuged and 1 ml 90% methanol was added to permeabilize the cells followed by an incubation for overnight (ON) at −20C. Samples were washed 2x with PBS containing 0.5% BSA. 100–100 ul of p-Tyr and IgG Isotype control antibodies were added and incubated at room temperature (RT) for 1 h. The p-Tyr antibody and IgG Isotype control antibodies (Cell Signaling, 9411, 5415) were diluted and used at equal concentrations (1 ug/ml). After incubation and washing with PBS containing 0.5% BSA, 100 ul PE-conjugated donkey anti-mouse antibody (1:200 dilution, Jackson Immuno Research, 715–116–150) was added per sample and incubated for 30 min at RT. Samples were washed with PBS containing 0.5% BSA then resuspended in PBS. Measurement was performed on BD Accuri™ C6 Cytometer. Analysis and figures were prepared using FlowJo (FlowJo, LLC, Oregon).

### Calcium influx measurements on FACS

RIN-5AH beta cells were seeded (2 × 10^6^ cells) on 10 cm plates and incubated ON. Cells were loaded with 3 μM Fluo-4 AM (Molecular Probes, F14201) with addition of a final amount of 0.02% Pluronic-F127 (Molecular Probes, P6867) for 45 min at 37C, in 5% CO_2_. After washing with HBSS (Life Technologies) without calcium and magnesium (HBSS−) cells were trypsinized then washed again with HBSS−. Cells were resuspended in 1 ml HBSS containing calcium and magnesium (HBSS++) or HBSS− before starting the measurements. Base fluorescent signal was recorded for 30 s in FL-1 channel. Gaps on FL-1/time graphs indicate the addition of compounds. Then samples were measured continuously for the desired time. Unstained cells were used as negative controls. To validate our assay we used the SERCA inhibitor Thapsigargin in HBSS− and the Ca^2+^ ionophore A23187 and GBA in HBSS++ buffers. Non-stained cells treated with compounds were used as negative control (Supplementary Fig. 7a.). Calcium influx measurements could not be carried out with sunitinib because of its autofluorescent nature. Compounds used in the calcium influx experiments were purchased from various sources (Chloroquine diphosphate - Sigma, C6628; Quinidine - Sigma, Q3625; Verapamil - Santa Cruz, sc-3590A; Efonidipine HCl monoethanolate - Sigma, E0159; Glibenclamide - Santa Cruz, sc-200982; Gatifloxacine - Santa Cruz, sc-204762; Levofloxacine HCl - Santa Cruz, sc-202693; Thapsigargin - Serva, 36000; A23187 Calcium ionophore - Sigma, C7522). Quantitative evaluation was done by gating every 5 s of mean fluorescence levels in the FL-1/time graphs (Supplementary Fig. 7b.). AUC was calculated from 50s-325s, baseline was set for the first untreated 35 s. Data were acquired on BD-FACSCalibur flow cytometer, data visualizations and MFI was evaluated with FlowJo software. AUC calculations were prepared in GraphPad Prism 7.00.

### Spinning disc confocal microscopy

RIN-5AH beta cells were plated (30.00 cells) in 35 mm u-Dishes (IBIDI, 80136) and incubated overnight at 37 °C, in 5% CO2. Cells were loaded with Fluo-4 AM for 45 min at 37C, 5% CO2 then washed 2x with HBSS−. Afterwards HBSS++ was added and base fluorescence signal was recorded for 30 s. Treatments were performed by using a perfusion pump (Ismatec) and final concentrations of 5 μM for compound **9** and 250 μM for glibenclamide. Monitoring and recording of fluorescence intensity was carried out on an UltraVIEW Vox spinning disk microscope system (PerkinElmer), which was attached to a HAMAMATSU ImagEM camera (cooled down to −80 °C). Calculation of results was done in Volocity software (PerkinElmer).

### Autofluorescence

Fluorescent signal of 5 μM compound **9** was recorded in aqueous solution using 0,1% DMSO and compared to fluorescein in order to rule out autofluorescent activity (data not shown).

### Patch clamp

RIN-5AH cells were trypsinized, centrifuged and resuspended in medium (RPMI-1640 containing 10% FBS and 2 mM glutamine). Cells were transferred into a recording chamber and allowed to settle down before beginning a slow perfusion with the external buffer (138.0 mM; NaCl; 5.6 mM KCl; 11.1 mM glucose; 1.2 mM; MgCl2; 2.6 mM CaCl2; 10.0 mM HEPES). Cells were sealed and patched by applying a gentle negative pressure. The components of intracellular solution were: 185 mM potassium-gluconate (Sigma); 13 mM HEPES; 13 mM NaCl; 1 mM MgCl2; 1 mM ATP (Sigma); 1 mM GTP (Sigma). Holding membrane potential was set to −70 mV for voltage clamp experiments. After patching the cells, for at least 10 s the baseline signal was recorded. Local application of drugs onto the cells from a thin glass capillary (similar to the patch-clamp pipette) was done using a Picospritzer II instrument (Parker) with a pressure of 5 psi and a pressure pulse lasting for 30 s. Different cells were used in voltage clamp and current clamp experiments. Each experiment was repeated 3–4 times and the total recording time was set to 2–4 minutes. Graphs were prepared in GraphPad Prism 7.00.

### Western Blot

1.2 × 10^6^ RIN-5AH beta cells were seeded in 6 well plates and incubated overnight in 37 °C and 5% CO_2_. Cells were washed 1x with PBS and treated for the indicated times. In combinatorial treatments, cells were pre-treated with inhibitors for 30 min, followed by a 10 min treatment with compound **9**. Cells were then washed with ice cold PBS and lysed in CelLytic M (Sigma, C2978) lysis buffer supplemented with aprotinin (4 μg/ml), NaF (10 mM), PMSF (1 mM), NaOV (1 mM), Phosphatase inhibitor cocktail 2 (Sigma, P5726). Lysate was collected by using cell scrapers. Protein concentration was determined by BCA assay kit according to user’s manual. 10–20 μg of protein was loaded in 9–15% SDS-gels. Gel electrophoresis was performed at 180 V. Proteins were transferred into nitrocellulose membrane (GE) by wet blotting for 1 h 15 min and 400 mA. Membranes were blocked in TBST containing 5% BSA (Sigma) for 1 h at RT following an overnight incubation at 4 °C with primary antibodies. Next day HRP-conjugated secondary antibody was applied for 1 h at RT. After incubation with antibodies, membranes were washed 3 × 15 min with TBST. Luminescent signal was developed with Enhanced ChemiLuminescent (ECL) Kit (PerkinElmer/NEN, NEL104001EA), signal was developed by using Hyperfilm TM MP (GE). Membranes were stripped in strip buffer (62.5 mM Tris/HCl pH 6.8; 2% SDS; 100 mM beta-mercaptoethanol) at 50 °C for 1,5 h and protein loading controls were checked by using total protein antibodies or alpha tubulin. The following antibodies were used: p-MEK1/2; p-ERK1/2; p-Tyr and p-Thr (Cell Signaling); pSer (Alexis); p-CAMKII (Abcam); ERK1/2 (Life technologies); a-tubulin (Sigma); Goat Anti-Mouse IgG secondary Ab (Jackson Immuno Research); Goat Anti-Rabbit IgG Ab (Biorad).

### Intracellular ATP level detection

20.000 cells were plated on flat bottom non-transparent black 96 well plates (Costar). Treatment was performed after overnight incubation for the desired time. Medium was removed and 80 ul CellTiter-Glo component (Promega) was added in a 1:1 volume to the wells, then plates were incubated at RT in dark for 15 min. Luminescence intensity was measured on TECAN Evolution microplate reader.

### Measurement of BrdU incorporation

RIN-5AH cells per well were seeded on 96 well plates and incubated overnight at 37 °C and 5% CO_2_. Treatment was performed in 100 ul per well for 48 h and 72 h. BrdU assay was analyzed by using the kit from Cell Signaling Technology (5492) and it was performed according to the user’s manual. In brief: 10 ul of 10x BrdU was added 24 h in advance to each sample and incubated at 37 °C and 5% CO_2_ until further steps. Luminescence was detected on TECAN Evolution microplate reader.

### Apoptosis analysis by Flow Cytometry

Apoptosis was detected by using Annexin V FITC and PI doublestaining (Thermo Scientific, V13242). 300.000 cells were plated on 6 well plates and incubated overnight at 37 °C and 5% CO_2_. Cells were treated with the compounds for the desired time. Cells were washed with ice cold PBS and trypsinized for 3 min. Then ice cold PBS was added to each sample and centrifuged. Samples were resuspended in 100 ul annexin binding buffer and staining was performed according to user’s manual. Samples were stored on ice in annexin binding buffer until measurement. A total of 20.000 ungated events were acquired on an Accuri C6 Plus Flow Cytometer. Data was analyzed in CFlow data acquisition and analysis software.

### Competitive kinase binding assay

Compound 9 and sunitinib were tested against 392 non-mutant and 59 mutant kinases in a competitive binding assay at 5 μM using DiscoverX KINOMEscan^®^ technology. Bosutinib was screened against 386 non-mutant and 56 mutant kinases. Results are reported in %Ctrl values, the lower values refer to stronger hits. We set the cutoff to 35%Ctrl to compare the target profiles of sunitinib, bosutinib and compound **9** (for more details see www.discoverx.com).

## Additional Information

**How to cite this article:** Orfi, Z. *et al*. Novel members of quinoline compound family enhance insulin secretion in RIN-5AH beta cells and in rat pancreatic islet microtissue. *Sci. Rep.* **7**, 44073; doi: 10.1038/srep44073 (2017).

**Publisher's note:** Springer Nature remains neutral with regard to jurisdictional claims in published maps and institutional affiliations.

## Supplementary Material

Supplementary Figures

Supplementary Table 1

Supplementary Table 2

## Figures and Tables

**Table 1:** Structure activity relationships (SAR) of quinoline compounds with (2,4-dichloro-5-methoxy-anilino) group at R4 position.

ID	R2	R3	R4	R6	R7	R8	Insulin (%)	Calcium (AUC)	pTyr (%)	Viability (%)
Compound 7	CN		(2,4-dichloro-5-methoxy-anilino)			Br	**116.1 ± 13.2**			
Compound 8	CN		(2,4-dichloro-5-methoxy-anilino)	OCH_3_	F		****115,8 ± 12	****89.5 ± 14	**66.0 ± 13.5**	**94.7 ± 6.7**
Compound 9	CN		(2,4-dichloro-5-methoxy-anilino)		F		****120.8 ± 6.6	****92.5 ± 12.8	**114.6 ± 22.26**	**84.7 ± 4.3**
Compound 10	CN		(2,4-dichloro-5-methoxy-anilino)	F			**89.4 ± 12.6**			
Compound 11	CN		(2,4-dichloro-5-methoxy-anilino)	OCH_3_			**78.2 ± 11.7**			
Compound 12		CN	(2,4-dichloro-5-methoxy-anilino)	OCH_3_	##		***74,0 ± 6	**5.3 ± 2,5**	*24.0 ± 3.8	*60.8 ± 16.7
Compound 13	CN		(2,4-dichloro-5-methoxy-anilino)			F	**69.9 ± 10.5**			
Compound 14	CN		(2,4-dichloro-5-methoxy-anilino)			CF_3_	**57.9 ± 14.1**			
Compound 15		CN	(2,4-dichloro-5-methoxy-anilino)	OCH_3_	F		**36,3 ± 3**	**9.5 ± 4.2**	**35.6 ± 6.7	**93.5 ± 5.1**
Compound 17	CONH_2_		(2,4-dichloro-5-methoxy-anilino)	OCH_3_			**33.6 ± 8.2**			
Compound 21	#		(2,4-dichloro-5-methoxy-anilino)	F			**20.4 ± 5.7**			
Compound 22	COOH		(2,4-dichloro-5-methoxy-anilino)	OCH_3_			**20.3 ± 4.8**			
Compound 32	CH_2_NH_2_		(2,4-dichloro-5-methoxy-anilino)		F		**−2,0 ± 10**	**3.4 ± 0.2**		**103.1 ± 3.3**
Compound 33	CN		(2,4-dichloro-5-methoxy-anilino)	F		Br	**−5.3 ± 5.5**			
Compound 34	CH_3_		(2,4-dichloro-5-methoxy-anilino)	OCH_3_			**−7.9 ± 2.3**			
Bosutinib		CN	(2,4-dichloro-5-methoxy-anilino)	OCH_3_	###		**14,0 ± 2.8**	**7.1 ± 1.4**	***18.6 ± 1.0	**94.0 ± 5.8**

Insulin is represented in % compared to DMSO treated cells (DMSO = 0%), Calcium influx is represented in AUC calculated from 50s–325 s (taking the period from 0–35 s as baseline), phopsho-tyrosine levels (pTyr) are indicated in % compared to DMSO treated cells (DMSO = 0%), viability values represent the % of healthy cells after treatments, compared to DMSO (where DMSO = 100%). All measurements indicated in the table were performed in RIN-5AH beta cells (n ≥ 3; SEM; t-test or ANOVA).

^#^Methanesulfonamidomethyl.

^##^3-aminopropoxy.

^###^3-(4-methylpiperazin-1-yl) propoxy.
