# Supplementary material for: Novel members of quinoline compound family enhance insulin secretion in RIN-5AH beta cells and in rat pancreatic islet microtissue
Source: Sci Rep. 2017 Mar 8;7:44073. doi: 10.1038/srep44073 (PMC5341024; doi:10.1038/srep44073)
Supplement: Supplementary Figures [file srep44073-s1.pdf]

Supplementary Materials

Novel members of quinoline compound family enhance insulin secretion in RIN-5AH beta cells and in rat pancreatic islet microtissue

Z. Orfi[1*], F. Waczek[2], F. Baska[2], I. Szabadkai[2], R. Torka[1], J. Hartmann[3], L. Orfi[2,4], A. Ullrich[1]

[1]Department of Molecular Biology, Max-Planck Institute of Biochemistry, Martinsried, Germany

[2]Vichem Chemie Research Ltd., Budapest, Hungary

[3]Institute of Neuroscience, Technische Universität München, Biedersteiner Str. 29, 80802, Munich, Germany

[4]Department of Pharmaceutical Chemistry, Semmelweis University, Budapest, Hungary

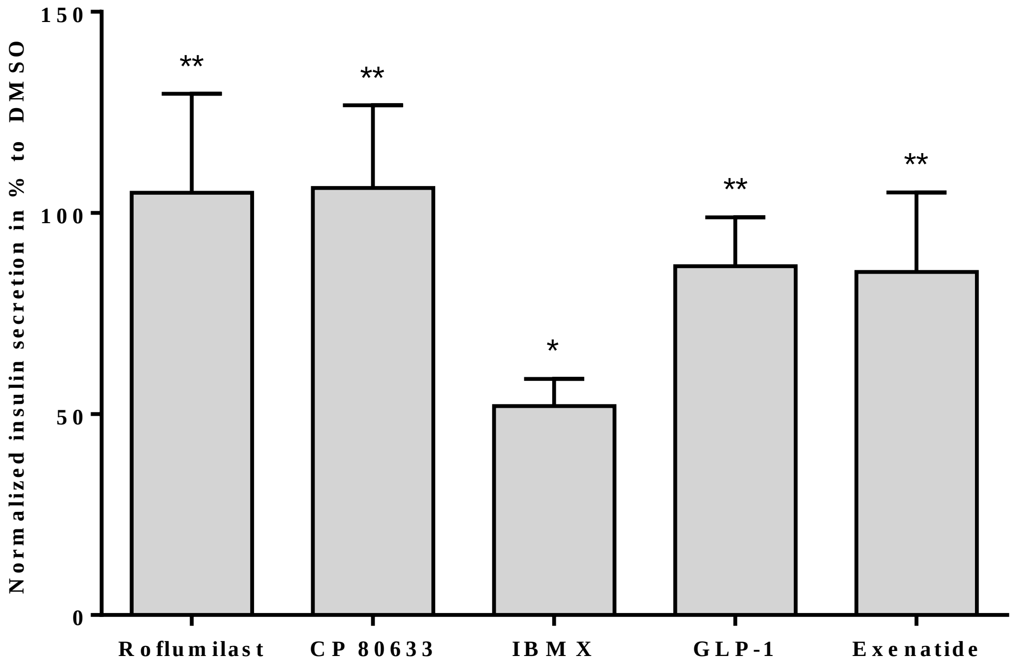

**Conc.**	**5 µM**	**5 µM**	**5 µM**	**0.3 µM**	**0.24 µM**
**Mean ± SEM**	**105 ± 24.7**	**106.2 ± 20.6**	**52 ± 6.8**	**86.7 ± 12.2**	**85.3 ± 19.8**
**p value**	**0.0012**	**0.0012**	**0.0379**	**0.0045**	**0.0045**

Supplementary Figure 1. Additional insulin secretagogue compounds induced insulin secretion in RIN-5AH beta cells. Roflumilast and CP 80633: PDE4 inhibitors, IBMX: pan-PDE inhibitor; GLP-1 and Exenatide: GLP-1 agonists (n=3-4; SEM; ANOVA/Holm Sidak; *p<0.05;**p<0.01)

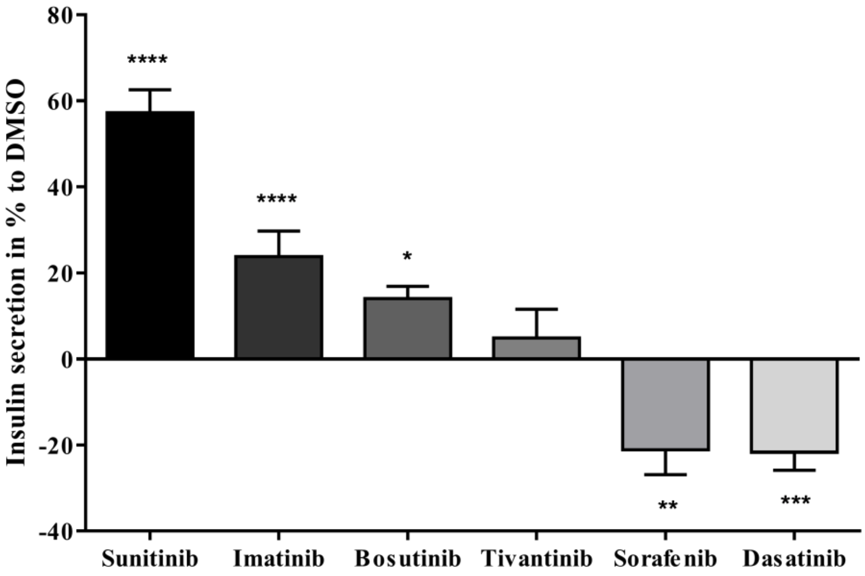

Supplementary Figure 2. Insulinotropic effect of some commercially available TKIs in RIN-5AH beta cells. Sunitinib produced a superior effect over other kinase inhibitors. Cells were treated at 5 µM for 2 h (n=5-10; SEM; One-way ANOVA / Dunnett; *p<0.05; **p<0.01; ***p<0.005; ****p<0.0001)

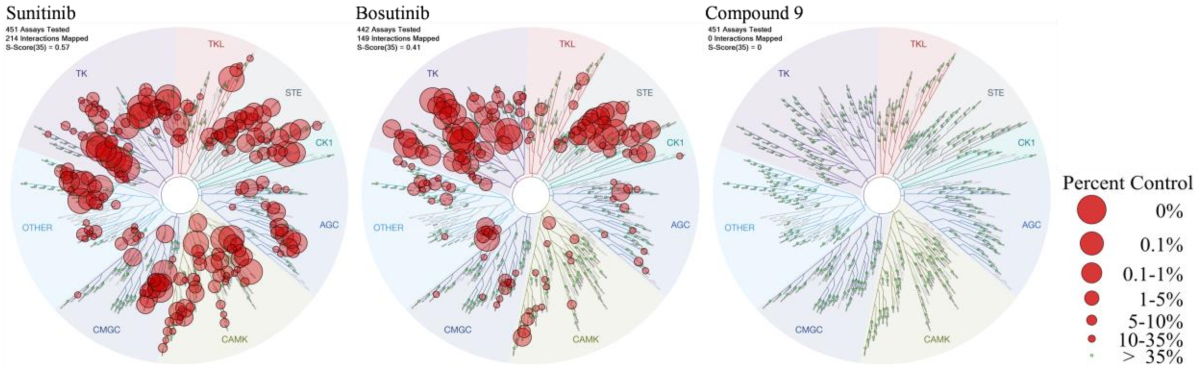

Supplementary Figure 3. Demonstrating of kinase hits detected in the competitive kinase binding assay. Evidently compound 9 didn't bind any kinases compared to bosutinib and sunitinib by using the same conditions for the assay (threshold set to 35 %Ctrl at 5 μM). S-Score(35) stands for selectivity , calculated by dividing the number of kinases bound by the total number of tested non-mutant kinases at 35 %Ctrl threshold. Images generated using TREE*spot*™ Software Tool and reprinted with permission from KINOME*scan*®, a division of DiscoveRx Corporation, © DISCOVERX CORPORATION 2010

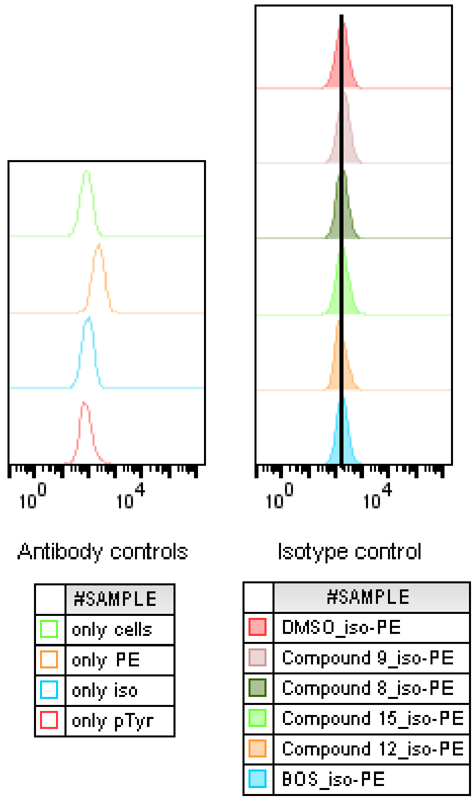

Supplementary Figure 4. Representative graphs of control samples used for the determination of pTyr levels by FACS. Experiments were repeated 3 times and gave similar results. (Iso=isotype control antibody, PE=Phycoerythrin labeled secondary antibody, pTyr=Total phospho-tyrosine antibody, BOS=bosutinib)

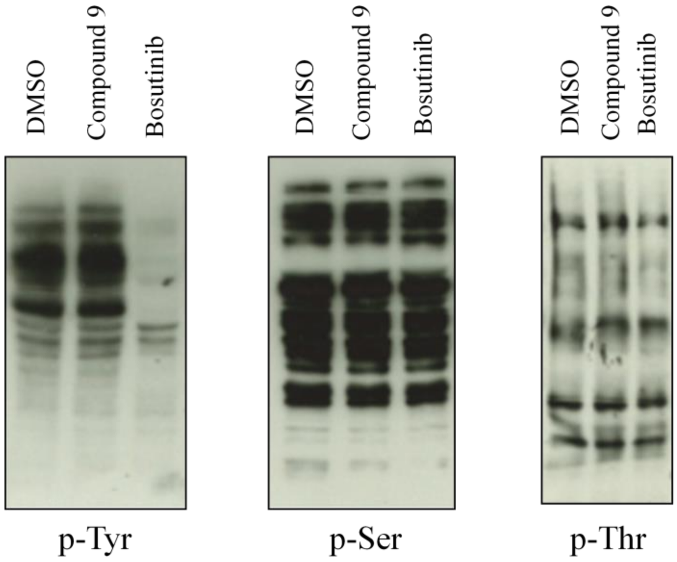

Supplementary Figure 5. There was no pTyr, pSer and pThr inhibition detected for Compound 9. RIN-5AH cells were analyzed by Western Blot results with non-specific pTyr, p-Ser and p-Thr antibodies. Compound treatments: 5 μM; 2 h

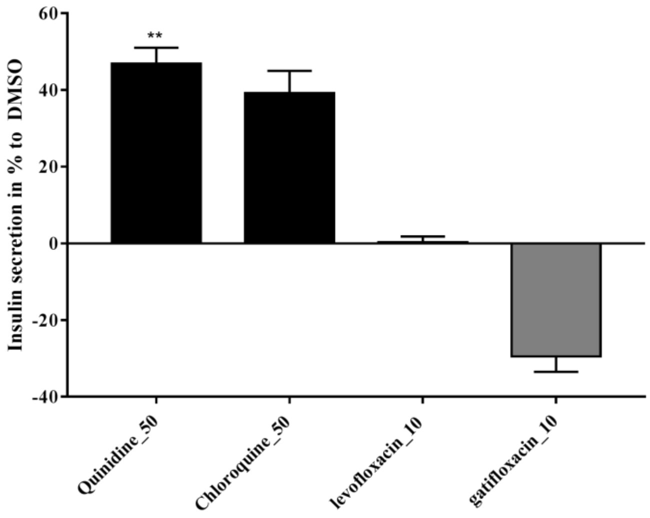

Supplementary Figure 6. Stimulation of insulin secretion by the quinoline derivatives quinidine and chloroquine in RIN-5AH beta cells. Additionally, levofloxacine and gatifloxacin was also tested since they were reported to have a K_{ATP} inhibitory property. In our experiments they didn't increase insulin level significantly. Concentrations are indicated in µM. (n=3; SEM; One-way ANOVA/Tukey; **$p<0.01$)

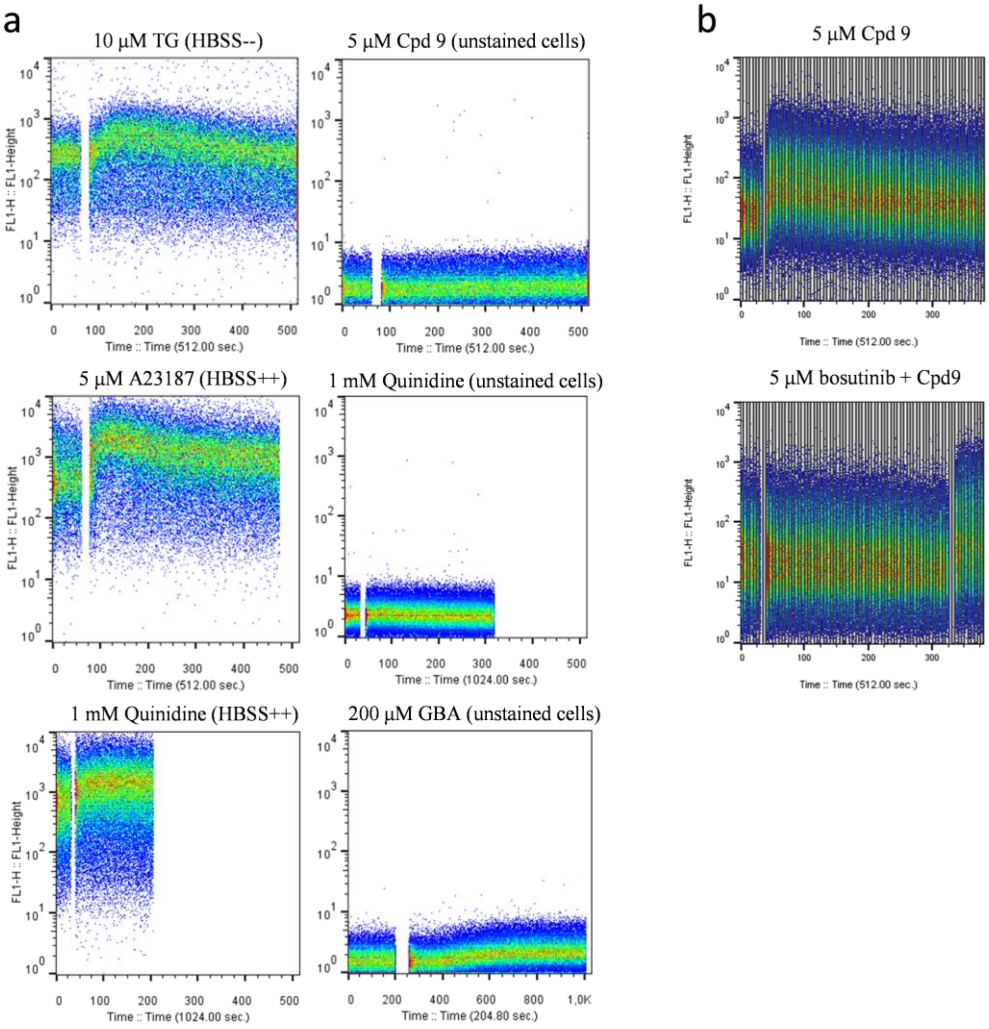

Supplementary Figure 7. Additional representative examples and reference controls used for Ca2+ influx measurements in RIN-5AH beta cells on FACS. (**a**) Thapsigargin (TG) was used as positive control in Ca^{2+}/Mg2+ free environments, and A21387 were used for experiments in Ca2+/Mg2+ containing buffer. Quinidine increased calcium influx only at 1 mM concentration. Unstained cells were used as negative controls in the assay. (**b**) Illustration of evaulation of the active compound 9 (Cpd9) and the inactive compound (bosutinib), 5s gates are displayed on the graphs. Cpd9 increased calcium influx. No calcium influx was detected by applying bosutinib on the cells. To validate cell viability and responsiveness, Cpd9 was employed as a second treatment, which could increase [Ca]$_i$. All measurements $n \geq 3$.

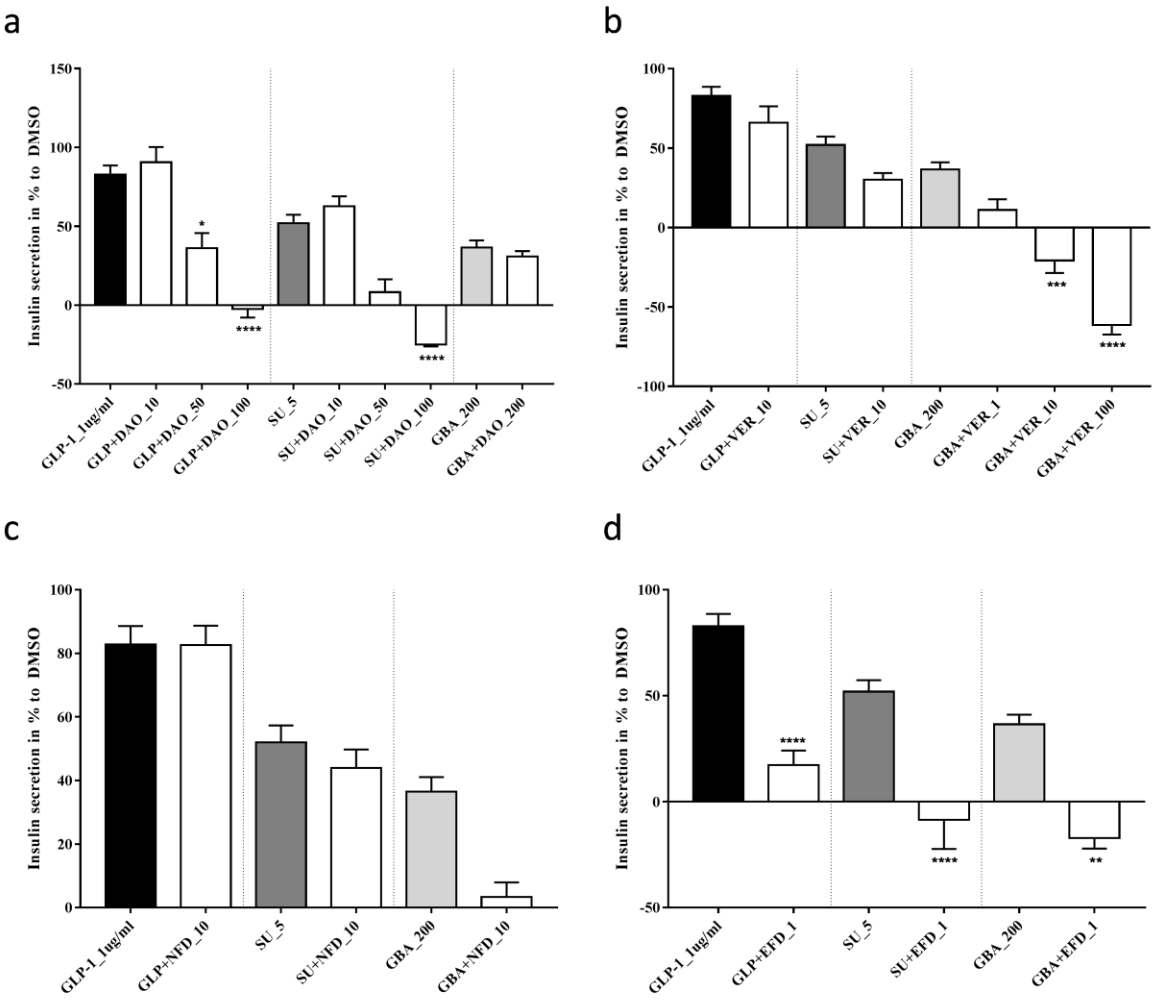

Supplementary Figure 8. Combinatorial treatments (empty bars) with GLP-1 (black), sunitinib (SU, grey) and glibenclamide (GBA, light grey). (**a**) Combinations with KATP opener diazoxide (DAO). (**b** and **c**) Combinations with L-type Ca2+ channel inhibitors, verapamil (VER) and nifedipine (NFD). (**d**) Combinations with L-/T-type channel inhibitor efonidipine (EFD). Values after compound names indicate concentrations in μM. (n=4-6; SEM; One-way ANOVA/Tukey; significancy was calculated by comparing individual drug treatments to their combination treatments *p<0.05;**p<0.01;***p<0.005;****p<0.0001)

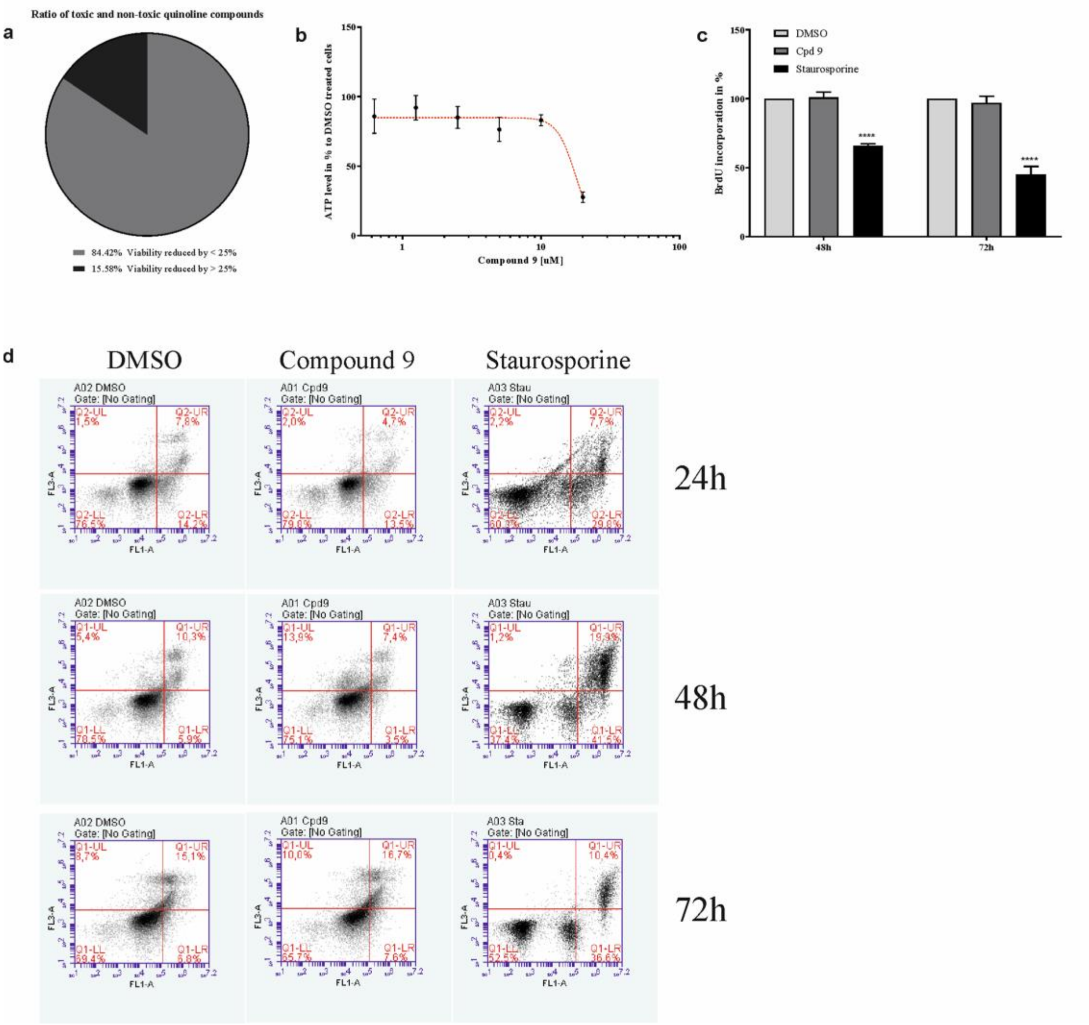

Supplementary Figure 9. Effects of quinoline derivatives on cell viability. **(a)** Quinoline compounds were tested for cell viability (CellTiter-Glo), including bosutinib. 12 compounds decreased the viability after 72 h and 5 μM treatment by at least 25%, 65 compounds didn't reduce the cell viability by more than 25% (n=3). **(b)** Dose response curve of compound 9 treated RIN-5AH cells, analyzed by CellTiter-Glo assay. Cell viability was disturbed above 10μM. **(c)** BrdU incorporation is displayed in % to DMSO treated cells (staurosporine = 2.5uM; n=3, ANOVA, ****p<000.1). **(d)** Representative graphs of apoptotic/dead cell analysis by propidium iodide and annexin V FITC double staining. Compound **9** didn't induce apoptosis in RIN-5AH cells (n=3). 2.5uM staurosporine was used as positive control in the experiments **(c and d)**.
